# Mediators of Obesity Do Not Influence SARS-CoV-2 Infection or Activation of Primary Human Lung Microvascular Endothelial Cells *In Vitro*

**DOI:** 10.3389/fimmu.2022.879033

**Published:** 2022-06-28

**Authors:** Bram M. ter Ellen, Jelmer Niewold, Antine Flikweert, Anneke C. Muller Kobold, Peter Heeringa, Matijs van Meurs, Jolanda M. Smit, Peter H. J. van der Voort, Izabela A. Rodenhuis-Zybert, Jill Moser

**Affiliations:** ^1^ Department of Medical Microbiology and Infection Prevention, University of Groningen, University Medical Center Groningen, Groningen, Netherlands; ^2^ Department of Critical Care, University of Groningen, University Medical Center Groningen, Groningen, Netherlands; ^3^ Department of Pulmonary Medicine, Amphia Hospital, Breda, Netherlands; ^4^ Department of Laboratory Medicine, University of Groningen, University Medical Center Groningen, Groningen, Netherlands; ^5^ Department of Pathology and Medical Biology, University of Groningen, University Medical Center Groningen, Groningen, Netherlands

**Keywords:** endothelial activation, SARS-CoV-2, adipokines, inflammation, leptin, endothelial cells

## Abstract

Clinical observations have shown that obesity is associated with the severe outcome of SARS-CoV-2 infection hallmarked by microvascular dysfunction in the lungs and other organs. Excess visceral fat and high systemic levels of adipose tissue (AT) derived mediators such as leptin and other adipokines have also been linked to endothelial dysfunction. Consequently, we hypothesized that AT-derived mediators may exacerbate microvascular dysfunction during of SARS-CoV-2 infection and tested this in a primary human lung microvascular endothelial (HLMVEC) cell model. Our results indicate that HLMVEC are not susceptible to SARS-CoV-2 infection since no expression of viral proteins and no newly produced virus was detected. In addition, exposure to the virus did not induce endothelial activation as evidenced by a lack of adhesion molecule, E-selectin, VCAM-1, ICAM-1, and inflammatory cytokine IL-6 induction. Incubation of endothelial cells with the pro-inflammatory AT-derived mediator, leptin, prior to virus inoculation, did not alter the expression of endothelial SARS-CoV-2 entry receptors and did not alter their susceptibility to infection. Furthermore, it did not induce inflammatory activation of endothelial cells. To verify if the lack of activated phenotype in the presence of adipokines was not leptin-specific, we exposed endothelial cells to plasma obtained from critically ill obese COVID-19 patients. Plasma exposure did not result in E-selectin, VCAM-1, ICAM-1, or IL-6 induction. Together our results strongly suggest that aberrant inflammatory endothelial responses are not mounted by direct SARS-CoV-2 infection of endothelial cells, even in the presence of leptin and other mediators of obesity. Instead, endothelial activation associated with COVID-19 is likely a result of inflammatory responses initiated by other cells. Further studies are required to investigate the mechanisms regulating endothelial behavior in COVID-19 and the mechanisms driving severe disease in obese individuals.

## Introduction

Since the emergence of severe acute respiratory syndrome coronavirus 2 (SARS-CoV-2) in, 2019, the virus has infected over 270 million people and resulted in 5,3 million deaths worldwide (1), causing a tremendous burden on human health. SARS-CoV-2 infection in humans can lead to various manifestations ranging from asymptomatic disease to mild flu-like symptoms to severe lethal disease (2). Patients with severe coronavirus disease, 2019 (COVID-19) can develop acute respiratory distress syndrome (ARDS), which can lead to multiple organ failure. The development of severe disease is mainly caused by pulmonary injury induced by direct viral infection of the lungs and the subsequent local immune responses trying to control and neutralize viral infection (3, 4).

Multiple risk factors and co-morbidities are associated with the development of severe COVID-19. Clinical observations have identified that most patients with severe respiratory failure admitted to the ICU are overweight or obese and have extensive visceral and subcutaneous adipose tissue (AT) (5–8). Both visceral and subcutaneous fat can produce AT-derived mediators, such as the proinflammatory adipokine leptin. Excess adipose tissue and associated local and systemic adipokines in obese patients can cause a chronic pro-inflammatory state predisposing them to thrombosis and other endothelial disturbances. It is therefore hypothesized that this chronic inflammatory state might exacerbate the immune responses to SARS-CoV-2 infection in obese patients rendering them susceptible to severe disease (8, 9). In line with this hypothesis, recent studies found plasma leptin to be increased in COVID-19 patients admitted to the intensive care (8, 10, 11).

A dysregulated immune response which is characterized by the release of pro-inflammatory cytokines is thought to be the leading cause of endothelial activation and dysfunction in severe COVID-19 patients (12–15). Both direct infection of endothelial cells and increased levels of leptin or other systemic adipokines could contribute to the endothelial activation and dysfunction observed in obese patients with severe COVID-19 (12–14). However, whether endothelial cells can be infected by SARS-CoV-2 and thereby contribute to endothelial dysfunction is still debated since contradicting evidence has been found (16–18). Furthermore, if and how, high levels of leptin or other AT-derived mediators could influence SARS-CoV-2 infection and endothelial activation is still unknown.

In this study we sought to elucidate how endothelial cells respond to direct SARS-CoV-2 exposure and if adipokines and other AT-derived mediators influence infection and endothelial activation *in vitro*. First, we investigated whether endothelial cells were susceptible to SARS-CoV-2 and if infection would lead to endothelial activation. Secondly, we examined if exposure of the adipokine, leptin would prime the endothelial cells promoting SARS-CoV-2 viral infection and endothelial activation. Lastly, since leptin is not the only systemic adipokine abundantly present in the blood of obese individuals, we investigated endothelial activation to plasma obtained from obese critically ill COVID-19 patients.

## Materials and Methods

### Cell Culture

Primary human lung microvascular endothelial cells (HMVEC-L/HLMVEC) (cat: #CC-2527, Lonza, Breda, The Netherlands) and Human umbilical vein endothelial cells (HUVEC) (cat: #CC2519, Lonza) were cultured in EBM-2 supplemented with EGM-2 endothelial growth SingleQuot kit supplement & growth factors (Lonza). All experiments were performed using passage 6 for HMVEC-L and passage 5 for HUVECs. The African green monkey Vero E6 cell line (ATCC CRL-1586) was cultured in Dulbecco’s minimal essential medium (DMEM) (Thermo Fisher Scientific, Waltham, MA, U.S.A), high glucose supplemented with 10% fetal bovine serum (FBS) (Life Science Production), 1% penicillin (100 U/mL), and 1% streptomycin (100 U/mL) (Gibco- Thermo Fisher Scientific). All cells were maintained at 37°C under 5% CO2 conditions.

### Virus Production and Characterization

The SARS-CoV-2 strain NL/2020 was obtained from European Virus Archive global (EVAg - 010V-03903). The original stock was passaged twice in Vero E6 cells to obtain a working stock. Infectious virus titers were determined by plaque assay on Vero E6 cells and defined as the number of plaque forming units (PFU) per mL. Briefly, Vero E6 cells were seeded at a density of 1.3×10^5^ cells/well in a 12-well plate format. At 24 hour post-seeding, cells were infected with 10-fold serial dilutions of the virus stock performed in duplicates. At 2 hours post inoculation (hpi), the wells were overlaid with 1% SeaPlaque agarose (Lonza) prepared in 2x MEM. Plaques were counted after 72 hpi. One plaque in the lowest dilution corresponds to 150 PFU/mL and was set as the detection limit of the assay.

### Patient Plasma

Plasma was collected for clinical purposes from 12 critically ill patients with severe COVID-19, and 4 COVID-19-negative patients admitted to the UMCG intensive care unit (ICU) during the first wave of COVID-19 admissions. The diagnosis of COVID-19 was confirmed by RT-PCR of oropharyngeal and nasopharyngeal swabs. Heparinized blood was centrifuged (1300 RCF for 10 minutes at 4°C) and stored at -80°C until need for experiments. Some samples were taken serially during hospitalization in either the ward (pre-ICU) and subsequently in the ICU. The median time from hospitalization to sample collection was 20 days (range 2-34 days). All patients required invasive mechanical ventilation in the ICU. Analyses were performed using residual plasma samples obtained from hospitalized patients for clinical purposes and is therefore not considered clinical research with human subjects as meant in the Medical Research Involving Human Subjects Act (WMO) (UMCG METc, 2020/492).

### SARS CoV-2 Infection

#### Direct SARS-CoV-2 Infection

HMVEC-Ls were infected at a confluency of 80% which comprises to around 1.2x10^5^ with SARS-CoV-2 at a multiplicity of infection (MOI) of 5 unless indicated otherwise. In experiments testing virus production, the inoculum was removed at 2 hpi, the cells washed twice with PBS and fresh medium subsequently added and incubation continued for 6 or 22 hr. Cells were harvested using trypsin at 8 and 24hpi and processed for flow cytometry or mRNA analysis. Cell-free supernatants were also collected, aliquoted, snap-frozen in liquid nitrogen, and stored at -80°C. Cells treated with 1µg/mL LPS (E. coli, serotype O26:B6, Sigma Aldrich, St. Louis, MO, USA) for 24 hours was used as a positive control for endothelial activation.

#### SARS-CoV-2 Infection in the Presence of Leptin

HMVEC-L were preincubated with recombinant human Leptin (Cat#: 398-LP, R&D Systems, Abington, U.K) for 16h prior to infection with SARS-CoV-2 MOI 5. For experiments using patient plasma, endothelial cells were incubated with 30%, 10% or 3.3% plasma diluted in EGM-2 media for 16 hours prior to infection. Cells were harvested using trypsin at 8 and 24 hpi and subjected to further analyses. Cell-free supernatants were collected, aliquoted, snap-frozen in liquid nitrogen and stored at -80°C until further analyses.

#### Frequency of Infection Analysis

Harvested cells were permeabilized using permeabilization (perm) buffer (PBS 1X (Ca/Mg free) 0.5% Tween) for 15 minutes at 4°C and subsequently incubated with primary monoclonal mouse anti-NSP8 antibody (GeneTex, Irvine, CA, USA) or monoclonal mouse anti-Spike antibody (GeneTex) diluted 1:500 in permeabilization buffer for 30 min 4°C. Cells were washed and subsequently stained with secondary rabbit anti-mouse AF647 antibody (Thermo Fisher Scientific) diluted 1:1000 in perm buffer for 30 min at 4°C in the dark. Stained cells were analyzed using a NovoCyte Quanteon (Agilent Technologies, Amstelveen, The Netherlands) flow cytometer, and data analyzed using Kaluza software (Beckman Coulter, Woerden, The Netherlands).

#### Progeny Virus Titrations

Infectious virus titer was determined using the plaque assay as described above. Levels of SARS-CoV-2 RNA in the supernatant were determined using RT-qPCR. Briefly, viral RNA was isolated from supernatants using the QIAmp Viral RNA Mini Kit (Qiagen) according to manufacturer’s protocol. CDNA synthesis from viral RNA was performed using Omniscript RT kit (Qiagen) with the reverse primer CARATGTTAAASACACTATTAGCATA. qPCR is performed by means of using the Qiagen Hot star Taq polymerase kit in combination with the primers GTGARATGGTCATGTGTGGCGG forward, CARATGTTAAASACACTATTAGCATA reverse and RdRp_SARSr-P2 (5’FAM/3’BHQ) probe CAGGTGGAACCT CATCAGGAGATGC. DNA amplification was performed for 50°C 120sec, 95°C 90sec and subsequently [95°C 15sec, 60°C 60sec] 40x.

### Gene Expression Analysis of Endothelial Genes

Total RNA was isolated from harvested cells using the RNeasy Plus Mini Kit (Qiagen, Venlo, The Netherlands) following the manufacturer’s protocol. RNA concentration (OD 260) and purity (OD260/OD280) were determined using a NanoDrop^®^ ND-1000 UV-Vis spectrophotometer (NanoDrop Technologies, Rockland, ME, USA). Samples with an OD260/OD280 ratio of ≥1.8 were included in the analysis. cDNA synthesis was performed as previously described (19). qPCR was performed using a ViiA 7 PCR system (Applied Biosystems, Nieuwerkerk aan den IJssel, The Netherlands) using the following assay-on-demand primers (Applied Biosystems), GAPDH (assay ID Hs99999905_m1), ACE2 (Hs01085333_m1), TMPRSS2 (Hs01122322_m1), CD147 (BSG) (Hs00936295_m1), Nrp1 (Hs00826128_m1), E-selectin (Hs00174057_m1), VCAM-1 (Hs00365486_m1), ICAM-1 (Hs00164932_m1), IL-6 (Hs00174131_m1), IL-8 (Hs00174103_m1).

### Protein Expression of Endothelial Adhesion Molecules

Harvested cells were fixed with 4% paraformaldehyde (PFA), washed with FACS buffer; 1x PBS (Gibco), 2% FBS (BioWhittaker) and subsequently stained for 30 min at 4°C using the following antibodies diluted in FACS buffer directed to endothelial adhesion molecules: CD62E E-selectin PE (1:100, clone HCD62E, #322606), CD106 VCAM-1 APC (1:100, clone STA, #305810) and CD54 ICAM-1 FITC (1:100, clone HCD54, #322720). Isotype-matched controls PE anti-mouse IgG2a (1:100, clone RMG2a-62, #407107), APC anti-mouse IgG1 (1:100, clone RMG1-1, #406609) and FITC anti-mouse IgG1 (1:100, clone RMG1-1, #406605) and used for setting positive cell gates. Cells were subsequently analyzed using a NovoCyte Quanteon (Agilent Technologies) flow cytometer, and data analyzed using Kaluza software (Beckman Coulter).

### Statistical Analysis

Statistical analysis of results was performed by one-way ANOVA followed by Bonferroni *post hoc* analysis to compare multiple replicate means using GraphPad Prism software v.9 (La Jolla, CA, USA). Differences were considered significant when p < 0.05.

## Results

### Lung Microvascular Endothelial Cells Are Not Susceptible and/or Permissive to SARS-CoV-2 Infection

**Figure 1:**
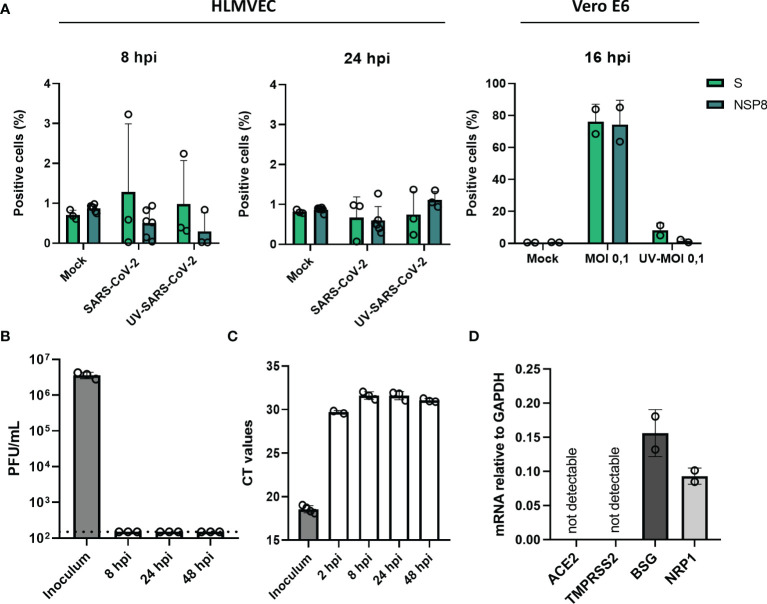
SARS-CoV-2 does not infect HLMVEC. HLMVEC were inoculated with SARS-CoV-2 at MOI 5 for 8, 24 and 48 hr and Vero E6 cells were inoculated for 16 hr. **(A)** Percentage of infected cells was determined in HLMVEC 8 and 24 hpi and Vero E6 cells 16 hpi by the protein expression of NSP8 and S determined by flow cytometry. The production of new infectious virus **(B)** and viral RNA **(C)** was determined at 8, 24 and 48 hpi by plaque assay and qPCR respectively. The dotted line indicates the threshold of detection. Data are represented as mean ± SD of at least three independent experiments. Each symbol represents data from a single independent experiment. **(D)** The mRNA levels of ACE2, TMPRSS2, BSG and NRP1 were obtained by RT-qPCR. Gene expression values were normalized to the expression of the housekeeping gene GAPDH. Student T tests were used to evaluate statistical differences and a p value ≤ 0.05 was considered significant with *p ≤ 0.05, **p ≤ 0.01 and ***p ≤ 0.001. In the absence of ‘*’ the data is non-significant.

To determine if endothelial cells are susceptible to SARS-CoV-2 infection, HLMVEC were infected with SARS-CoV-2 at an MOI of 1, and MOI 5 for 8 and 24 hr. Infection was determined by detecting the expression of SARS-CoV-2 nonstructural protein 8 (NSP8) and Spike (S) protein expression by flow cytometry. We did not detect NSP8 or Spike protein in endothelial cells at both 8 and 24 hr post-infection (hpi) at MOI 1 (**Supplemental Figure 1**) and MOI 5 (**Figure 1A**), indicating that HLMVEC are not infected by SARS-CoV-2. In contrast, NSP8 and S proteins were detected in SARS-CoV-2 infected Vero E6 control cells (**Figure 1A**). Confirming these findings, we determined the presence of secreted infectious particles in the cell supernatant but detected no PFUs 24 hpi in the supernatant SARS-CoV-2 (**Figure 1B**). In addition, we determined the levels of viral RdRp RNA after washing 2 hpi. The 2 hpi wash sample had a CT value of ≈30 whereas the CT values for viral RNA at 8, 24 and 48 hpi did not decrease and were higher ≈ 31 than the CT value of the leftover inoculum ≈ 30 (**Figure 1C**). In line with these findings, we found undetectable mRNA levels of the main SARS-CoV-2 cell entry mediators, angiotensin-converting enzyme 2 (ACE2) receptor and transmembrane protease serine 2 (TMPRSS2), yet alternative receptors basigin/CD147 (BSG) and Neuropilin-1 (NRP1), were expressed by HLMVEC (**Figure 1D**). Collectively these results show that HLMVEC are not susceptible and/or permissive to SARS-CoV-2 infection which might be related to the low expression of ACE2 and TMPRSS2.

### Lung Microvascular Endothelial Cells Do Not Elicit an Inflammatory Response to SARS-CoV-2 *In Vitro*

**Figure 2:**
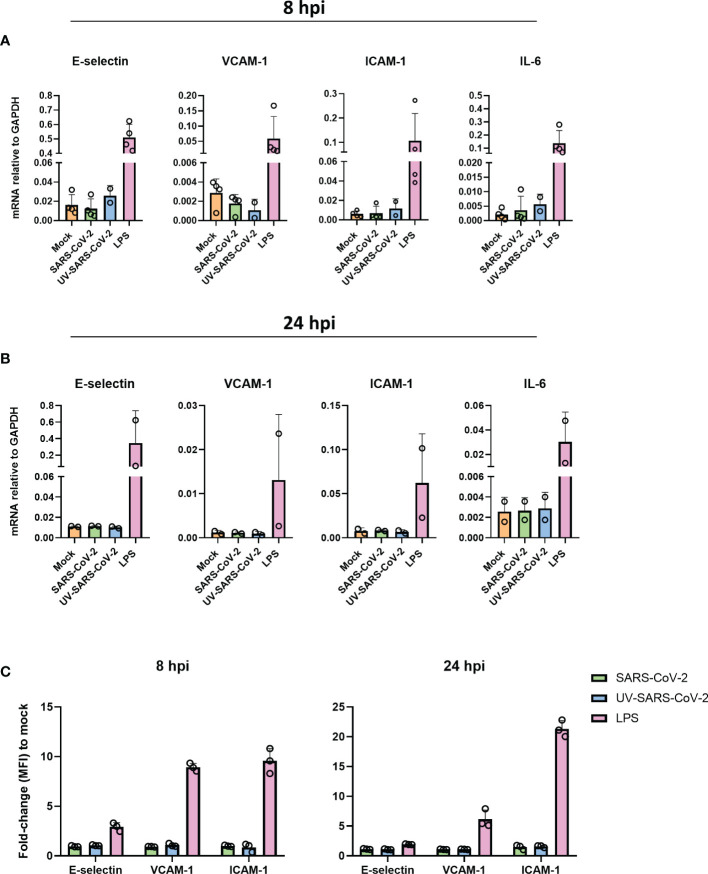
SARS-CoV-2 does not activate HLMVEC. **(A, B)** HLMVEC were inoculated with SARS-CoV-2 at MOI 5 or LPS 1 µg/mL for 8 or 24 hr. The mRNA levels of E-selectin, VCAM-1, ICAM-1, and IL-6 at 8 **(A)** and 24 hpi **(B)** were obtained by qPCR. Results are expressed as the mean ± SD of 2-4 individual experiments with duplicate technical replicates **(C)** Protein expression of E-selectin, VCAM-1 and ICAM-1 at 8 and 24 hpi by flowcytometry, data is represented as MFI fold change to mock and represented as the mean ± SD of at least three independent experiments. Each symbol represents data from a single independent experiment. Gene expression values were normalized to the expression of the housekeeping gene GAPDH. Student T tests were used to evaluate statistical differences and a p value ≤ 0.05 was considered significant with *p ≤ 0.05, **p ≤ 0.01 and ***p ≤ 0.001. In the absence of ‘*’ the data is non-significant.

We have shown that SARS-CoV-2 is unable to infect endothelial cells. Yet, SARS-CoV-2 particles could still be sensed by endothelial cells and thereby elicit an inflammatory response. Moreover, inflammatory endothelial responses are hypothesized to play a crucial role in the progression of respiratory failure and associated coagulative complications (12–14). To investigate this, we inoculated HLMVEC with SARS-CoV-2 MOI 1 and MOI 5 or the equivalent volume of UV-inactivated SARS-CoV-2 for 8 and 24 hr and determined the mRNA expression of endothelial activation markers, E-selectin, VCAM-1, and ICAM-1, as well as inflammatory cytokine IL-6. The mRNA levels of all genes did not differ in response to SARS-CoV-2 or UV-inactivated SARS-CoV-2 at 8 hpi (**Figure 2A**) and 24 hpi (**Figure 2B**) compared to the mock, whereas the positive control LPS upregulated E-selectin, VCAM-1, ICAM-1 and Il-6 in HLMVEC at both 8 and 24 hpi (**Figures 2A,B**). Moreover, E-selectin, VCAM-1 and ICAM-1 protein levels in HLMVEC remain unchanged by exposure to SARS-CoV-2 for 8 and 24 hpi determined by flow cytometry (**Figure 2C**). In contrast, LPS was able to induce upregulation of all endothelial adhesion molecules (**Figure 2C**). All together, these findings show that HLMVEC are unable to elicit a direct inflammatory activation response to SARS-CoV-2.

### Leptin Does Not Facilitate Infection nor Promote Activation of Lung Microvascular Endothelial Cells *In Vitro*

**Figure 3:**
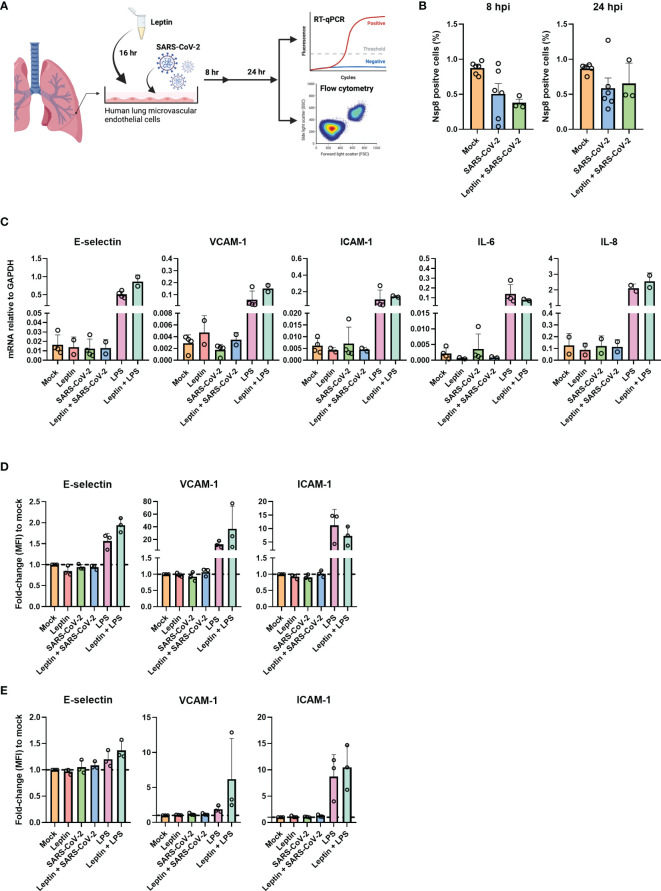
Leptin does not facilitate infection or activation of HLMVEC. **(A)** schematic overview of the experimental set-up. HLMVEC were pre-incubated or with leptin, 1000ng/mL or with media for 16 hr. Cells were then inoculated with SARS-CoV-2 at MOI 5 or LPS 1µg/mL for 8 and 24 hr. **(B)** Percentage of infected cells were determined at 8 and 24 hpi by flowcytometry. **(C)** Gene expression of E-selectin, VCAM-1, ICAM-1, IL-6, and IL-8 was determined by qPCR at 8 hpi. Results are expressed as the mean ± SD of 2-4 individual experiments done with duplicate technical replicates **(D, E)** Cells were analyzed for protein expression of E-selectin, VCAM-1 and ICAM-1 at 8 **(D)** and 24 hpi **(E)** by flowcytometry. Data are represented as MFI fold-change to mock and represented as mean ± SD of at least three independent experiments. Each symbol represents data from a single independent experiment. Student T test was used to evaluate statistical differences and a p value ≤ 0.05 was considered significant with *p ≤ 0.05, **p ≤ 0.01 and ***p ≤ 0.001. In the absence of ‘*’ the data is non-significant. **Figure 3A** was created with Biorender.com.

Clinical observations have found that the severity of COVID-19 is associated with obesity (5, 6). High levels of plasma leptin and adipokines are directly associated with the extent of obesity and have previously been shown to facilitate endothelial dysfunction (5, 20–22). Moreover, we and others reported before that plasma leptin levels are increased in critically ill COVID-19 patients (8, 10, 11). We therefore hypothesized that adipokines such as leptin may prime endothelial cells, thereby facilitating SARS-CoV-2 sensing and/or infection and driving the endothelial dysfunction which is characteristic of severe COVID-19. To determine dose dependent endothelial responses upon leptin exposure, we stimulated HUVEC with physiological and high concentrations of leptin, 100ng/mL and, 1000 ng/mL respectively. Incubation with both leptin concentrations did not result in endothelial activation, since protein expression of E-selectin, VCAM-1 and ICAM-1 was not increased (**Supplemental Figure 3A**). To verify this in HLMVEC we incubated the cells with the highest concentration of leptin. HLMVEC with leptin alone did not result in endothelial activation or inflammation since the mRNA levels of E-selectin, VCAM-1, ICAM-1, IL-6 and IL-8 were not upregulated compared to control (**Figure 3C**). To test if leptin incubation sensitized the cells to infection and/or activation, we preincubated HLMVEC with, 1000ng/mL recombinant leptin for 16 hr and subsequently inoculated the cells with SARS-CoV-2 MOI 5 or positive control LPS for 8 or 24 hr (**Figure 3A**). We found no NSP8 protein expression in the HLMVEC treated with leptin prior to inoculation at both 8 and 24 hpi (**Figure 3B**). In addition, the expression of SARS-CoV-2 entry receptors were also not influenced by leptin incubation. ACE2 and TMPRSS2 mRNA levels remained undetectable while BSG and NRP1 expression remained unchanged (**Supplemental Figure 3B**). Furthermore, the mRNA levels of endothelial activation and inflammation genes remained at mock control levels after leptin exposure with or without SARS-CoV-2 infection (**Figure 3C**). Similar to our mRNA findings, E-selectin, VCAM-1 and ICAM-1 protein levels were also not influenced by leptin, or leptin preincubation prior SARS-CoV-2 infection at 8 hpi (**Figure 3D**) and 24 hpi (**Figure 3E**). These results indicate that leptin alone does not influence endothelial inflammatory activation responses, nor does it promote endothelial cell infection, or modulate SARS-CoV-2-mediated endothelial activation.

### Plasma From Overweight and Obese Critically Ill COVID-19 Patients Does Not Activate Endothelial Cells *In Vitro*

**Figure 4:**
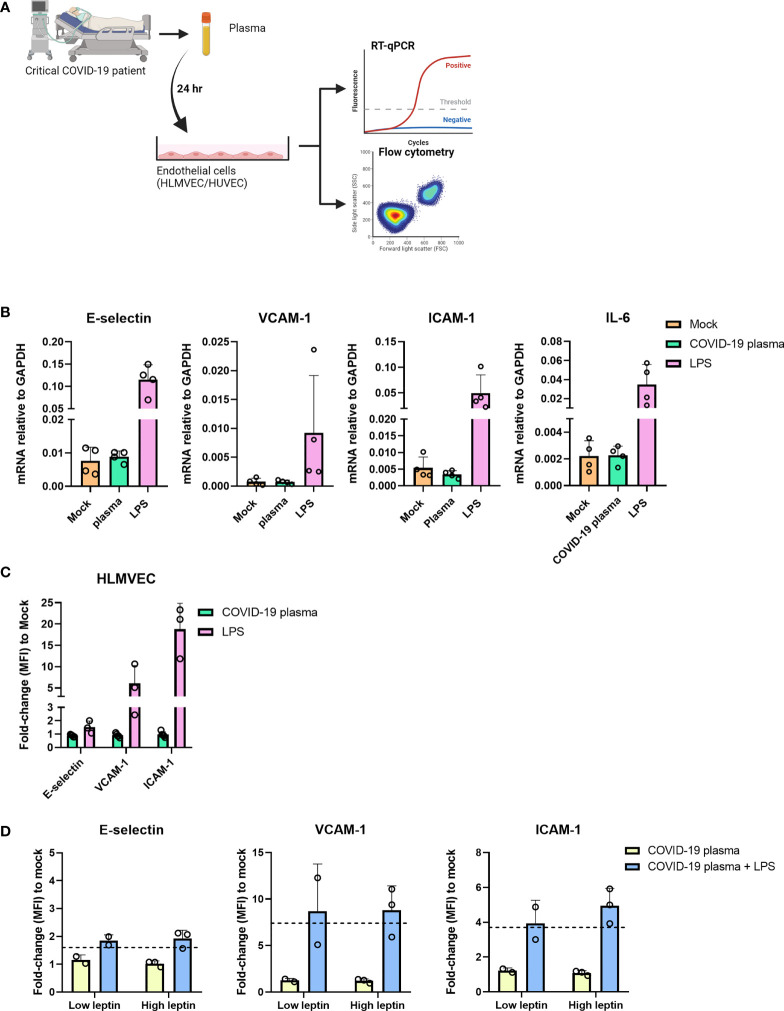
Plasma from severe COVID-19 patients does not activate HLMVEC. **(A)** schematic overview of the experimental set-up. HLMVEC were incubated with 30% plasma from critically ill COVID-19 patients or stimulated with LPS 1 µg/mL for 24 hr. **(B)** The mRNA levels of E-selectin, VCAM-1, ICAM-1, and IL-6 were determined by RT-qPCR. Results are expressed as the mean ± SD of 4 individual experiments with duplicate technical replicates **(C)** Protein levels of E-selectin, VCAM-1 and ICAM-1 were determined by flowcytometry, data is represented as MFI fold-change to mock and represented as mean ± SD of at least three independent experiments. **(D)** HUVECs were incubated with critically ill COVID-19 patient plasma containing high leptin concentrations n=3 donors (average 77,3 ng/mL) or low leptin concentrations n=2 donors (average 2,3 ng/mL) and supplemented with or without LPS 1 ug/mL for 4 hr. Protein levels of E-selectin, VCAM-1 and ICAM-1 were determined by flow cytometry, data is represented as the MFI fold-change to mock and represented as mean ± SD. Dotted line indicates fold change to mock of only LPS stimulated cells for 4 hr. Each symbol represents data from a single independent experiment. Student T test was used to evaluate statistical differences and a p value ≤ 0.05 was considered significant with *p ≤ 0.05, **p ≤ 0.01 and ***p ≤ 0.001. In the absence of ‘*’ the data is non-significant. **Figure 4A** was created with Biorender.com.

In the *in vivo* situation leptin is not the only adipokine or AT-derived mediator present in the blood of obese individuals (23). To investigate each known adipokine separately *in vitro* would be a tedious time-consuming approach. We therefore opted to investigate the influence of adipokines and AT-derived-mediators and proinflammatory cytokines on endothelial cells found in the plasma of patients admitted to the intensive care unit with severe COVID-19. We incubated HLMVEC for 24 hr with 30% patient plasma obtained from severe COVID-19 patients who were admitted to the ICU and analyzed the cells for the expression of E-selectin, VCAM-1, ICAM-1, and IL-6 (**Figure 4A**). We observed no differences in the mRNA levels of all genes between the mock and plasma incubated samples, whereas the positive control LPS upregulated all the analyzed genes (**Figure 4B**). The protein expression of E-selectin, VCAM-1 and ICAM-1 was also unaltered in samples incubated with COVID-19 patient plasma compared to mock (**Figure 4C**). Similar findings were observed in HUVEC incubated with different concentrations of patient plasma (**Supplemental Figure 4A**) or with COVID-19 negative ICU patient plasma (**Supplemental Figure 4B**). To exclude the possibility that the patient plasma contained anti-inflammatory mediators or traces of immune suppressive medication administered during their ICU stay that might prevent endothelial activation, we incubated the endothelial cells for 4 hr with plasma n=3 containing high leptin concentrations (average 77,3 ng/mL) and plasma n=2 containing low leptin concentrations (average 2,3 ng/mL) supplemented with or without LPS. Endothelial cells only became activated when they were exposed to patient plasma supplemented with LPS indicating that the plasma does not contain any immune suppressive medication or anti-inflammatory mediators that would prevent endothelial activation (**Figure 4D**). Together these results suggest that plasma from overweight and obese critically ill COVID-19 patients are not able to activate endothelial cells *in vitro* and that this effect is not due to anti-inflammatory mediators or immunosuppressive medication that might have been present in the plasma.

## Discussion

Severe obesity is a major risk factor for the development of severe COVID-19 with patients often requiring mechanical ventilation and other organ support in the ICU (5). Leptin, one of systemic adipokines increased in critically ill COVID-19 patients is known to promote endothelial dysfunction (24, 25) and coagulation (26, 27). To investigate if leptin might play a role in influencing the severe disease symptoms in obese COVID-19 patients, we determined whether endothelial cells were susceptible to SARS-CoV-2 infection and whether mediators of obesity such as leptin would promote endothelial activation *in vitro*. We found that both human lung and umbilical cord vascular endothelial cells did not replicate SARS-CoV-2 even at high multiplicities of infection. We also did not detect any evidence of virus sensing as exposure to the virus did not lead to endothelial activation. Notably, the presence of leptin or plasma from obese critically ill COVID-19 patients did not alter this phenotype.

High levels of leptin along with leptin resistance in obesity are not only responsible for establishing a proinflammatory state but also make obese individuals more prone to cardiovascular complications (28, 29). We therefore hypothesized that mediators of obesity such as high leptin levels might influence endothelial responses to SARS-CoV-2 infection. The presence of adipokines did not promote infection or replication of SARS-CoV-2 in endothelial cells. These findings are likely related to the low endothelial expression of ACE2 and TMPRSS2, which remained undetectable when cells were cultured in the presence of high concentrations of leptin. Notably, the also unaltered by the presence of leptin, expression of BSG and NRP1 receptors described to mediate SARS-CoV-2 entry (30, 31), did not confer susceptibility of the endothelial cells to the virus. This is in line with recent reports, suggesting that ACE2 and TMPRSS2 expression might be prerequisite for BSG and NRP1 to exert their infection-potentiating activity (32) Interestingly, previous studies have shown that ACE2 expression is regulated by IFNα and IFNγ signalling (33). Moreover, an *in vitro* study by Klouda et al. found that IFNα increased the expression of ACE2 in primary pulmonary endothelial cells (34). Surprisingly, despite a potent induction of ACE2 expression, IFNα treatment resulted only in an extremely low frequency of infected endothelial cells with on average 1 and 3% at the MOI of 1 and 5 respectively. For comparison, infection of ACE2 expressing primary nasal epithelial cells, MOI of 0.1 results in the infection of approximately 40% of cells (35). Taken together, the relatively low infection frequency in IFNα-treated endothelial cells suggest that although susceptible to SARS-CoV-2, they are not permissive to infection.

Interestingly, the spike protein of SARS-CoV-2 alone was shown to activate endothelial cells which was dependent on integrin α5β1 signaling (36). However, we found that endothelial exposure of the virus did not activate endothelial cells, suggesting limited ability of pattern recognition receptors expressed on endothelial cells to sense SARS-COV-2 *in vitro*. Previous *in vivo* studies have shown that obesity induced endothelial dysfunction and promotes acute lung injury (37, 38), however, these reports have recently been retracted due to issues regarding the validity of the results. Our results show that the presence of leptin either in physiological or high levels, did not induce endothelial inflammatory activation responses, nor did it prime the SARS-CoV-2-mediated endothelial activation. In addition to our observations, recent findings suggest that leptin in fact exerts beneficial effects protecting against endothelial activation and inflammation (39).

Klouda et al., also elegantly showed that when IFNα treatment was combined with other cytokines, mimicking COVID-19-induced systemic inflammation *in vivo*, the influence of IFNα on ACE2 expression in endothelial cells was lost (34). In line with these findings, we found that incubation of endothelial cells with COVID-19 patient plasma containing a mixture of cytokines including IFNα, adipokines and other mediators did not alter ACE2 expression and also not result in endothelial activation. In contrast to our findings, a recent study by Shi et al. using serum from a large cohort of COVID-19 patients found that the surface expression of E-selectin, VCAM-1 and ICAM-1 were around 2, 4 and 3-fold increase in HUVEC compared to control serum (40). However, the response of the serum on endothelial cells was very heterogenic with a large sub-population of patient samples not inducing endothelial activation. We used plasma and flow cytometry to determine the surface expression of the endothelial activation markers, whereas Shi et al., used serum and quantified the expression of surface endothelial markers using in-cell ELISA (40). These distinct techniques may account for the differences found between these studies. Together, our *in vitro* findings suggest that altered systemic leptin or other plasma adipokine levels might not be driving endothelial dysfunction associated with the severe organ manifestations in obese COVID-19 patients admitted to the ICU.

Although our results and those of others (17, 18) suggest that endothelial cells are unlikely to be infected with SARS-CoV-2 *in vivo*. We cannot exclude the possibility that endothelial cells within the organs of critically ill COVID-19 patients become infected with SARS-CoV-2. The results from autopsy studies are controversial and show viral particles in the close vicinity of the microvasculature (41, 42), but often it is unclear if specifically, the endothelium is infected. Having said that Liu et al., recently used multiple tools to demonstrate SARS-CoV-2 infection of the endothelium *in vivo* (16), whereas other recent studies report no infection of the endothelium (18). Whether endothelial cell infection of SARS-CoV-2 occurs *in vivo* remains a topic of debate. However, until now most studies conclude that the endothelium might become infected, but that it is not likely to be the primary or main site of SARS-CoV-2 infection in COVID-19 patients (18, 43). Alternatively, endothelial dysfunction can also be induced indirectly by immune hyperinflammatory responses or epithelial-endothelial cross talk after SARS-CoV-2 infection (44). Importantly, endothelial cell functions are to a certain extent dependent on the surrounding microenvironment such as the interactions with adjacent specialized cells (i.e., pericytes, podocytes, epithelial cells) and blood flow dynamics (45, 46). Endothelial cell gene signatures are rapidly lost when they are removed from their *in vivo* microenvironment and put into culture which may explain the lack of SARS-CoV-2 infection *in vitro* (45). We found the expression of endothelial cell ACE2 remained undetectable in cells which were cultured under flow-conditions (data not shown), and SARS-CoV-2 did not infect endothelial cells in 3D vessels under flow conditions (18). In this respect, perhaps future studies investigating endothelial responses in COVID-19 should move towards *in vivo* models, or by investigating post-mortem organs immediately after death ensuring intact viral RNA and pathology (47). Our research group has previously shown that laser microdissection of organ microvascular compartments is possible in murine models and human organ tissue (48, 49). This would allow us to identify if endothelial cells are indeed infected by SARS-CoV-2 in patients with COVID-19 and will allow transcriptomic analysis to investigate the dysregulated endothelial responses in severe COVID-19 patients giving an insight into the mechanisms involved.

Increased inflammation, endothelial activation and vascular permeability due to (in)direct endothelial infection of SARS-CoV-2 may cause edema, hemorrhage, and microvascular thrombosis, affecting gas exchange in the infected lungs as well as causing injury and functional defects in other organs such as the kidney (50). Analysis of longitudinal plasma samples have shown that soluble endothelial markers increase during the course of COVID-19 and sVCAM-1 specifically is associated with non-survival (51). This study and others illustrate the importance of endothelial responses in driving severe disease (43, 52, 53) as well as post-COVID-19 persistent lung damage (54).

Together our *in vitro* results suggest that aberrant inflammatory endothelial responses are not mounted by direct endothelial infection of SARS-CoV-2 even in the presence of leptin and other mediators of obesity. Further studies are required to investigate the mechanisms regulating endothelial dysfunction in COVID-19 and the mechanisms driving severe disease in obese individuals.

## Data Availability Statement

The original contributions presented in the study are included in the article/**Supplementary Material**. Further inquiries can be directed to the corresponding author.

## Author Contributions

JM, IR-Z, and PH designed the study. AF, AM, and PV provided plasma and clinical data. BE and JN performed the experiments and analyzed the data. PH, AM, MM, JS, and PV provided valuable input on the statistical analysis and interpretation of the results. BE, IR-Z, and JM wrote and edited the manuscript. All authors critically revised the manuscript and approved the submitted version.

## Funding

This work was supported by the ZonMw (Project Number: 10430012010006). BE was supported by GSMS of the RuG/UMCG.

## Conflict of Interest

The authors declare that the research was conducted in the absence of any commercial or financial relationships that could be construed as a potential conflict of interest.

## Publisher’s Note

All claims expressed in this article are solely those of the authors and do not necessarily represent those of their affiliated organizations, or those of the publisher, the editors and the reviewers. Any product that may be evaluated in this article, or claim that may be made by its manufacturer, is not guaranteed or endorsed by the publisher.
