# Supplementary figures and images for: Mediators of Obesity Do Not Influence SARS-CoV-2 Infection or Activation of Primary Human Lung Microvascular Endothelial Cells In Vitro
Source: Front Immunol. 2022 Jun 28;13:879033. doi: 10.3389/fimmu.2022.879033 (PMC9273911; doi:10.3389/fimmu.2022.879033)

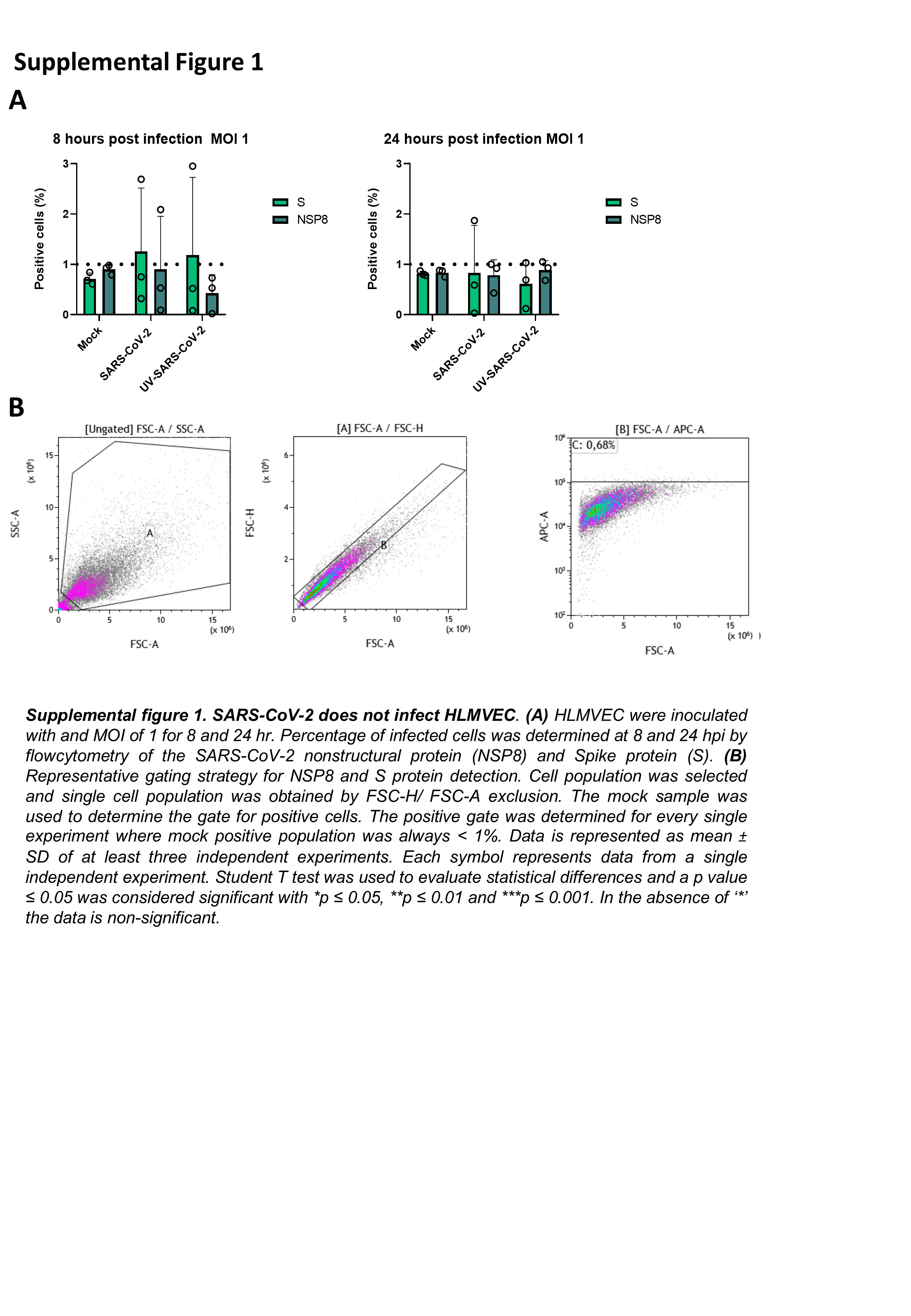

Supplement: Supplementary file 1 [file Image_1.tif]

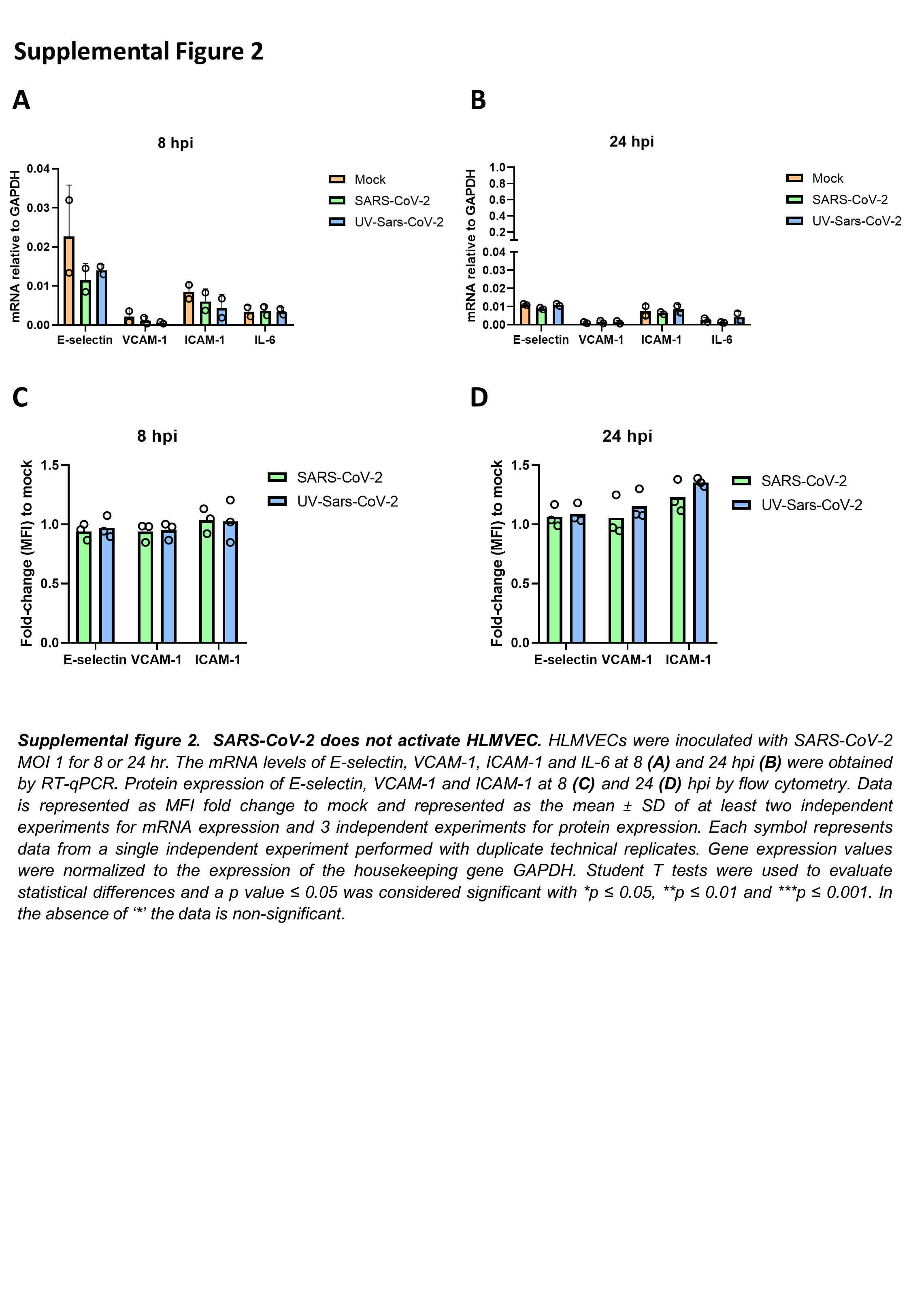

Supplement: Supplementary file 2 [file Image_2.tif]

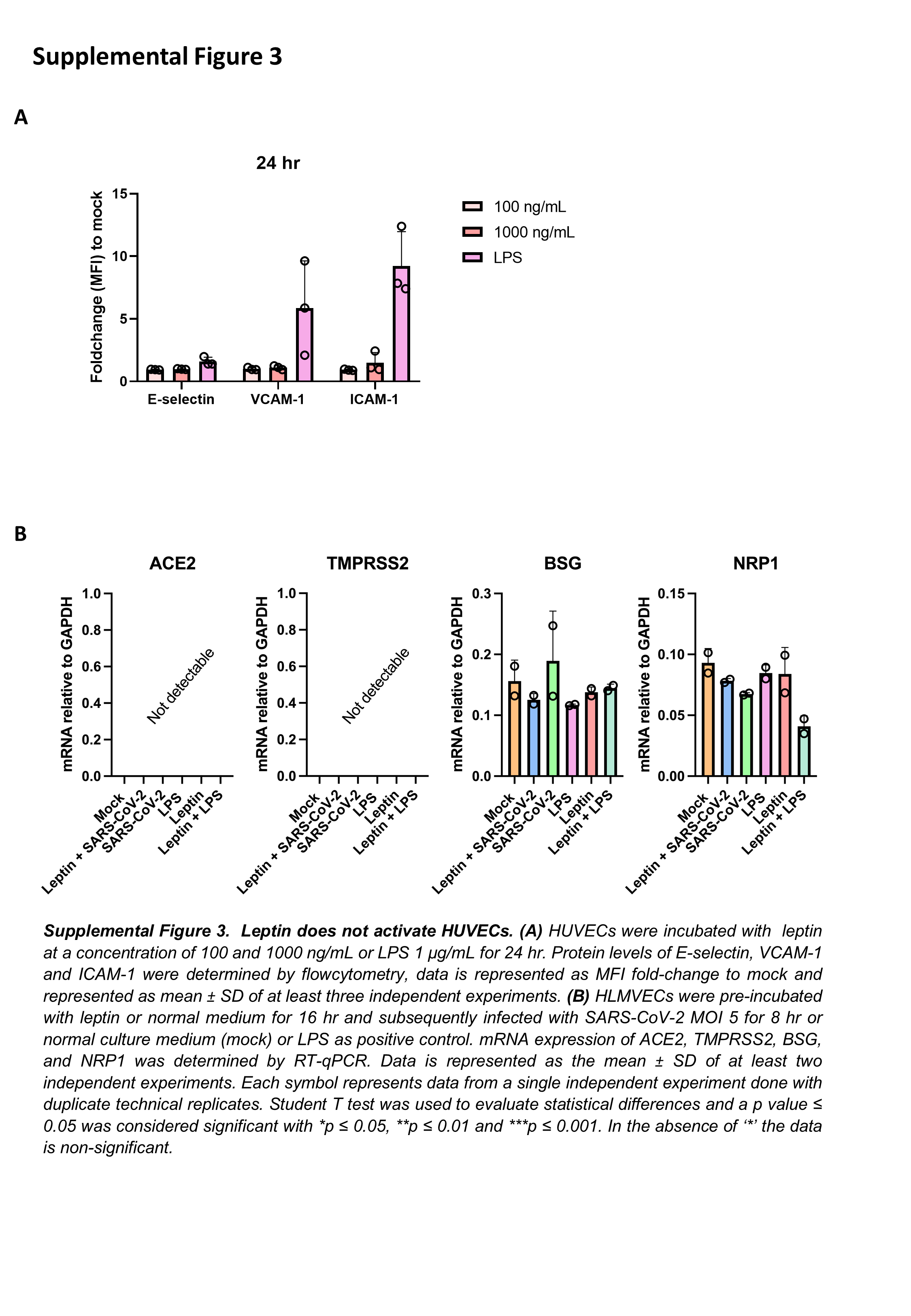

Supplement: Supplementary file 3 [file Image_3.tif]

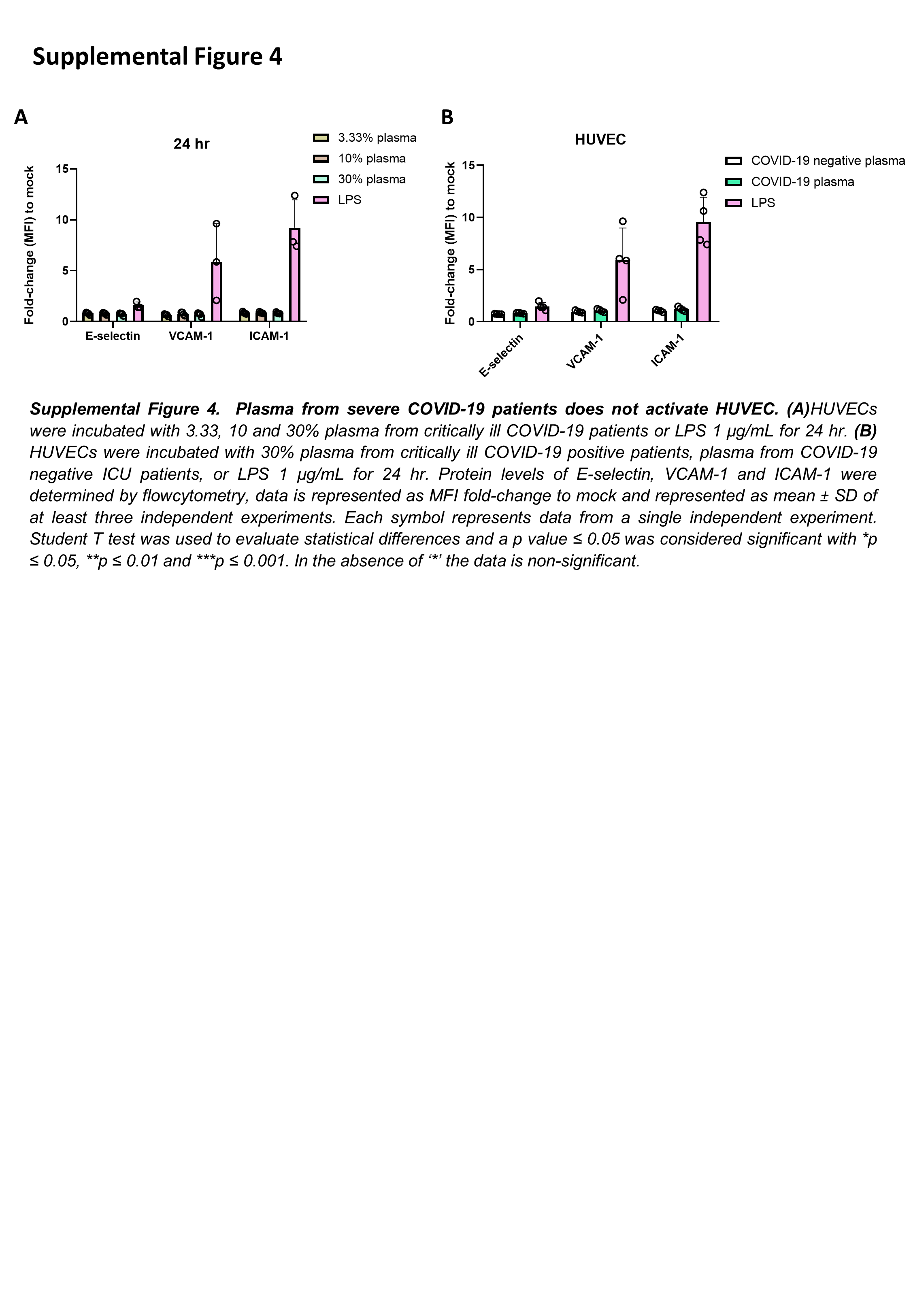

Supplement: Supplementary file 4 [file Image_4.tif]
